# Phytochemical and pharmacological investigation of the ethanol extract of *Byttneria pilosa* Roxb.

**DOI:** 10.1186/s40816-021-00333-w

**Published:** 2022-01-02

**Authors:** Liton Sikder, Md. Roich Khan, Shanita Zaman Smrity, Muhammad Torequl Islam, Shams Ara Khan

**Affiliations:** Department of Pharmacy, Life Science Faculty, Bangabandhu Sheikh Mujibur Rahman Science and Technology University, Gopalganj, Dhaka, 8100 Bangladesh

**Keywords:** *Byttneria pilosa*, Anti-inflammatory, Anti-nociceptive, Clot lysis

## Abstract

**Background:**

Traditionally, the herb *Byttneria pilosa* Roxb. is used for bone fractures, boils, scabies, rheumatalgia, snake bites, syphilis, elephantiasis, poisoning, and eye infection. Scientific reports suggest that it has significant anti-inflammatory, analgesic, anti-diarrheal, anxiolytic, locomotion, sedative and anti-obesity effects. This study aims at the investigation of the phytochemical and pharmacological properties of the ethanol extract of this herb.

**Methods:**

Fresh whole plant was extracted with absolute ethanol. A preliminary phytochemical investigation was followed by the evaluation of thrombolytic, anti-inflammatory, and anti-nociceptive activities by applying human clotted blood lysis, egg albumin, and acetic acid-induced writhing models, respectively.

**Results:**

Phytochemical investigation suggests that *B. pilosa* possesses alkaloids, flavonoids, glycosides, terpenoids, tannins, saponins, and reducing sugars. The extract exhibited clot lysis and anti-inflammatory effects in a concentration-dependent manner. *B. pilosa* extract at 250 and 500 mg/kg also showed significant (*p* < 0.05) dose-dependent anti-nociceptive activity in *Swiss* albino mice.

**Conclusion:**

The *B. pilosa* ethanol extract contains many important secondary metabolites and has thrombolytic, anti-inflammatory, and anti-nociceptive activities. More research is necessary on this hopeful medicinal herb.

## Introduction

Medicinal plants still remain a potential source of modern therapeutic agents for various diseases [51]. It may be due to their low side effects and high safety profile in experimental animals and humans [13]. Plant-derived compounds are beneficial for designing new chemical entities in the drug discovery and development processes. More than 60–70% of the antimicrobial and anticancer agents currently in clinical use are natural products or their derivatives [34]*.*

In the drug discovery and development context, the bioactivity of plants or their parts such as leaves, stems, and roots frequently used in traditional medicine is evaluated in laboratory-based bioassays [50]. In this context, plant-based lead compounds have become inspiration for the development of potential drugs [29]. Generally, medicinal plants or their derivatives are promising sources of alternative therapeutics [20] and can be used for some life-threatening diseases, including the novel coronavirus disease 2019 (Covid-19) [23].

**Fig. 1:**
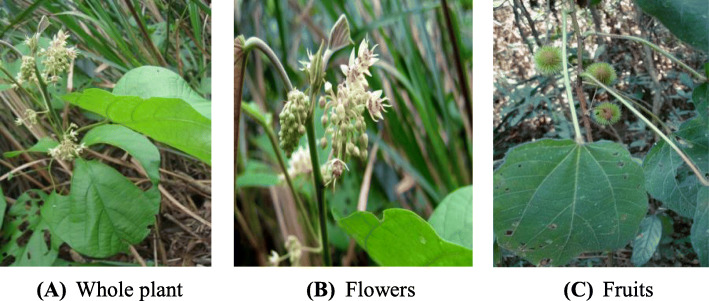
Morphological structure of *Byttneria pilosa* Roxb*.***A** Whole plant, **B** Flowers, **C** Fruits

*Byttneria pilosa* Roxb. (Malvaceae) (Fig. 1) is commonly known as Harjora in Bangladesh. It is found in the forests of Chittagong, Chittagong Hill Tracts, Cox’s Bazar, Sylhet, Srimangal, Gajni (Sherpur) and Habiganj districts of Bangladesh. It is an annual glabrous herb that can be grown as an annual, reproducing from the seeds [17]. The stems are large woody climbers with grooved, strigose, branchlets, and the leaves are sub or bicular, palmately 3-lobed, and pilose on both surfaces. The flowers of *B. pilosa* are minute campanulate, in a lax, much branched inflorescence, while the capsule is globose, the size of a large cherry, and studded with subulate barbed prickles. It becomes mature in the winter season.

*B. pilosa* is used by rural people as a traditional medicine. The paste prepared from the tender stem with leaves is tied around limbs for the treatment of fractured bones. The crushed stems and an infusion of the leaves are used to treat boils and scabies, respectively. The root paste is used to treat elephantiasis [22]. It is also used for rheumatalgia, snake bite, syphilis, poisoning, and eye infection [62]. A recent study suggests that this herb has significant anti-inflammatory, analgesic, anti-diarrheal, anxiolytic, locomotion and sedative effects in various test systems [22]. The authors also performed molecular docking, PASS prediction, and ADME analysis that suggests beta-sitosterol might be a potential candidate for these biological activities.

Our study was aimed at screening the phytochemical and pharmacological activities of the ethanol extract of *B. pilosa*. The results suggest that *B. pilosa* ethanol extract contains many important secondary metablies, including alkaloids, flavonoids, glycosides, terpenoids, tannins, and saponins. The extract showed clot lysis, anti-inflammatory, and anti-nociceptive activities in different test systems.

Plant-based molecular farming is cheap and relatively scalable [32]. Furthermore, they are generally safer because they do not pose the risk of product contamination with endotoxins, infectious viruses, or prions [46]. On the other hand, plants can perform complex post-translational modifications (e.g., Asn-linked (*N*) glycosylation), that can be further engineered to achieve humanized biomolecules [12]. Our current findings suggest that *B. pilosa* herb may be a hopeful alternative phyto-based therapeutic tool to manage many diseases in humans. However, the conclusion of this project has been drawn on the basis of limited non-clinical and pre-clinical studies on its crude extract. The active principles and their possible molecular interactions are yet to be discovered.

It is clear that the knowledge of ancient and traditional systems of medicine has now transcended to the modern pharmaceutical industry. Therefore, the focus of any phyto-pharmacological study is often to discover new therapeutic agents or lead compounds from medicinal plants. However, appropriate selection of target plant species, in this case, plays an important role. Interestingly, serendipity has played a significant role in plant-based drug discovery. For instance, the discovery of dicoumarol from fatal cattle poisoning by *Melilotus officinalis* was simply a serendipitous discovery [28], which then led to the development of the well-known anticoagulant warfarin. Conducting phyto-pharmacological research without any working hypotheses may lead to such a type of unexpected discovery. However, the chances of success are much poorer than with any targeted approach, and for this reason, we often rely on the basis of ethnopharmacological evidence and extensive literature activities on medicinal plants. Moreover, challenges, in this case, also lie in devising appropriate methods besides selecting the right plants that may fulfil these criteria. Knowling the overall facts, we have identified *B. pilosa* by an expert and selected some in vitro and in vivo assay methods for this study. Prior to this, we also gathered knowledge about its traditional and scientific evidence from reliable and authentic sources.

## Materials and methods

### Collection, identification, and extraction of plant materials

The fresh whole plant was collected from the forest of the University of Chittagong, Chittagong, Bangladesh in the month of January and was identified by the taxonomist at the Bangladesh National Herbarium, Dhaka. A voucher specimen was deposited with the accession no.: DACB 46758. After collection, the plant materials were shade dried (temperature not exceeding 45 °C) for 7 days. Then the dried materials were cut into small pieces and ground into coarse powder with the help of a suitable mechanical chopper. The powdered materials (250 g) were then soaked in 700 mL of absolute ethanol with occasional manual stirring for 15 days. Then the extract was finally filtered using Whatman filter paper. The organic solvent (ethanol) was evaporated with a rotary evaporator at room temperature to get the dried crude extract (yield value of 2.064%). Then the dried crude extract was stored in the refrigerator at 4 °C.

### Reagents and chemicals

Ascorbic acid and acetic acid were purchased from Merck, Germany, while tween-80 was purchased from Loba Chemie Pvt. Ltd., India. Solvents and all the other reagents and chemicals were of analytical grade. The diclofenac sodium was collected from Aristopharma Ltd., Bangladesh, while the standard antibiotic discs (Ciprofloxacin 5 μg/disc) were purchased from Oxoid Ltd., UK, and sterile blank discs were also purchased from BBL, Cocksville, USA. Streptokinase (Durakinase) powder for reconstitution; 1,500,000 units/vial used in the clot lysis test was purchased from Dong Kook Pharmaceutical Co. Ltd., South Korea.

### Experimental animals

Adult male albino mice (21–34 g), purchased from the animal resource branch of Jahangirnagar University, Dhaka, were used for the study. The animals were housed under standard environmental conditions (temperature: 25 ± 2 °C, humidity: 50 ± 5%, and 12 h light/dark cycles) in sanitized polypropylene cages containing sterile paddy husk as bedding. Animals were freely accessing standard pellets as a basal diet and water ad libitum. All the animals were acclimatized for 2 weeks before starting the study. The animals were randomized into experimental and control groups and the food was withdrawn 12 h before the experimental hours.

### Phytochemical investigation

Preliminary phytochemical group tests were done to investigate plant secondary metabolites such as alkaloids, glycosides, flavonoids, steroids, terpenoids, saponins, tannins, gums, and reducing sugars in the extracts by the methods of Harborne [19] and Trease and Evans [55].

### Anti-inflammatory activity test (egg albumin model)

To determine the anti-inflammatory effect of the crude extract, the egg albumin model was used [56] with a slight modification. In brief, the inhibition of protein denaturation indicates the anti-inflammatory effect of a substance (e.g., extract, drug). In this study, *B. pilosa* crude extract at 5–320 μg/mL was tested by taking distilled water and acetyl salicylic acid as negative and positive controls, respectively. The crude extract was prepared in a similar manner as mentioned in the previous test (clotlysis assay). The 5 mL reaction mixture consisted of egg albumin (0.5 mL, from fresh hen’s egg), phosphate buffered saline (3.5 mL) (pH 6.4) and varying concentrations of test sample (1 mL). Then the mixtures were incubated at (37 ± 2 °C) in a bio-oxygen demand (BOD) incubator for 15 min and then heated at 70 °C for 5 min. After cooling, the absorbance was measured at 660 nm (UV/VIS Sprectophotometer, Shimadzu, Japan) by using the vehicle as a blank. A similar volume of distilled water and acetyl salicylic acid (100 μg/mL) were used as negative and positive controls, respectively. The percentage of inhibition of protein denaturation was calculated by using the following formula:

$$ \%\mathrm{Inhibition}=\left[\left({\mathrm{Absorption}}_{\mathrm{Control}}-{\mathrm{Absorption}}_{\mathrm{Test}}\right)/{\mathrm{Absorption}}_{\mathrm{Control}}\right]\kern1em \times 100 $$

### Thrombolytic activity test

The venous blood drawn from healthy volunteers (*n* = 8) was distributed into the negative control (distilled water); standard (streptokinase); and 6 different concentrations (5 to 320 μg/mL) of the crude extract marked alpin tubes. For this purpose, the required amount of crude extract was dissolved in a small amount of ethanol, which was then reconstituted in distilled water qsto. Each tube was pre-weighed (W1) and contained 0.5 mL of blood. The tubes were then incubated at 37 °C for 45 min. After clot formation, serum was completely removed carefully (without disturbing the clot) and again weighed (W2) to determine the clot weight (W2-W1). After the addition of test sample/controls (100 μL/tube), the tubes were then re-incubated at 37 °C for 90 min. Then the lysed fluid was removed carefully and the tubes were weighed (W3) to observe the difference in weight after clot disruption (W3-W2). The difference obtained in weight taken before and after clot lysis was expressed as a percentage of clot lysis [45].

### Anti-nociceptive activity test (acetic acid induced writhing model)

This study was performed according to the method described by Koster et al. [27] and Ezeja et al. [15]. For this purpose, the experimental animals (*Swiss* mice) were randomly divided into 4 groups, namely negative control (Vehicle), positive control (Diclofenac sodium, 25 mg/kg), and 2 test groups (250 and 500 mg/kg). The control group received 0.5% tween-80 dissolved in a 0.9% NaCl solution. All the treatments were given via oral gavage at a dose of 10 mL/kg body weight. After 30 min of treatments, each mouse was treated with 0.7% acetic acid solution intra-peritoneally and the number of writhings provided by each animal was counted for 10 min.

### Statistical analysis

The results were presented as mean ± SEM. A student’s *t*-test was used to determine if there was a significant difference between the experimental and control groups. The test results were statistically significant when *p* < 0.05 at 95% confidence intervals. Half maximal effective concentrations (EC_50_s) were calculated by extraploting the test concentration vs. responses (non-linear method) using GraphPad Prism software (version: 6.0, San Diego, California, USA. copyright© 1994–1999), considering *p* < 0.05 at a 95% confidence interval.

## Results

### Phytochemical studies

Table 1 suggests that the crude ethanol extract of *B. pilosa* possesses alkaloids, glycosides, flavonoids, terpenoids, steroids, tannins, saponins, and reducing sugars. More intensity was seen in the case of terpenoids, followed by alkaloids, glycosides, tannins, saponins, and reducing sugars. Poor intensity was seen in the case of the flavonoids and steroids tests, while no intensity was observed for the gum test.

**Table 1:** Secondary metabolites in *B. pilosa* ethanol extract

Phytochemical groups	Test results
Alkaloids	**++**
Glycosides	**++**
Flavonoids	**+**
Terpenoids	**+++**
Steroids	**+**
Tannins	**++**
Saponins	**++**
Gums	**–**
Reducing sugars	**++**

Intensity: (−) → No, (+) → Low, (++) → Moderate, (+++) → Strong

### Anti-inflammatory activity

*B. pilosa* extract concentration-dependently inhibited protein denaturation in the test tubes. The extract exhibited the highest (67.53 ± 0.33%) inhibition of albumin denaturation at 320 μg/mL concentration. ASA at 5 to 100 μg/mL showed total inhibition of protein denaturation by 21.23 ± 0.23 to 72.78 ± 0.07%, while the extract of *B. pilosa* at 5 to 320 μg/mL showed total inhibition of protein denaturation from 12.73 ± 0.17 to 67.53 ± 0.33%. Thus, the standard ASA at 100 μg/mL exhibited a better inhibitory effect than the test sample. The half maximal effective concentrations (EC_50_s) calculated for the extract and ASA were 125.30 ± 0.11 and 34.08 ± 0.23 μg/mL, respectively (Table 2).

**Table 2:** Total inhibition of protein denaturation by the *B. pilosa* ethanol extract and the controls

Treatments		Egg albumin test	
		*TIPD*	*EC*_*50*_*(CI; R*^*2*^*)*
NC		–	–
ASA (μg/mL)	5	21.23 ± 0.23	34.08 ± 0.23 μg/mL (19.34–60.04 μg/mL; 0.87)
	10	27.31 ± 0.34	
	20	32.21 ± 0.17	
	40	52.43 ± 0.07	
	80	57.23 ± 0.43	
	100	72.78 ± 0.07	
*B. pilosa* (μg/mL)	5	12.73 ± 0.17	125.30 ± 0.11 μg/mL (69.30–226.60 μg/mL; 0.89)
	10	24.68 ± 0.43	
	20	36.49 ± 0.36	
	40	42.86 ± 0.27	
	80	48.05 ± 0.23	
	160	59.35 ± 0.17	
	320	67.53 ± 0.33	

Values are Mean ± SEM (*n* = 5)

*NC* negative control, *ASA* acetyl salicylic acid, *TIPD* total inhibition of protein denaturation, *EC*_*50*_ half-maximal effective concentration, *CI* confidence of intervals, *R*^*2*^ co-efficient of determination

### Thrombolytic activity

The extract showed significant (*p* < 0.05) clot lysis capacity in a concentration-dependent manner. The extract of *B. pilosa* at 10 to 320 μg/mL showed clot lysis capacity of 8.83 ± 0.20 to 51.32 ± 0.73%. The highest clot lysis of the extract was 51.32 ± 0.73% at 320 μg/mL. The standard, SK, and NC produced clot lysis of 81.08 ± 0.01 and 2.57 ± 0.18%, respectively. The EC_50_ calculated for the test extract was 217.10 ± 0.08 μg/mL [CI: 132.30–356.10 μg/mL; R^2^: 0.90] (Table 3).

**Table 3:** Clot lysis capacity of *B. pilosa* ethanol extract and the controls

Treatments		% of clotlysis	EC_**50**_ [CI, R^**2**^]
NC		2.57 ± 0.18	–
SK (100 μl (30,000 I.U.))		81.08 ± 0.01*	–
*B. pilosa* (μg/ml)	10	8.83 ± 0.20*	217.10 ± 0.08 μg/mL [132.30–356.10 μg/mL, 0.90]
	20	10.99 ± 0.44*	
	40	13.42 ± 0.39*	
	80	21.15 ± 1.15*	
	160	36.31 ± 0.03*	
	320	51.32 ± 0.73*	

Values are mean ± SEM (*n* = 3); *t*-student’s test at 95% confidence intervals

*EC*_*50*_ half-maximal effective concentration, *CI* confidence of intervals, *R*^*2*^ co-efficient of determination, *SK* streptokinase

^*^p < 0.05 when compared to the NC (negative control) group

### Acetic acid induced writhing test

The *B. pilosa* ethanol extract dose-dependently modulated the anti-nociceptive parameters in experimental animals. The extract at both doses (250 and 500 mg/kg, p.o.) and the standard drug, diclofenac sodium (25 mg/kg, p.o.) significantly (p < 0.05) modulated the anti-nociceptive parameters in the test animals in comparison to the NC group. *B. pilosa* extract at 500 mg/kg showed writhing and %protection of 16.20 ± 1.16 and 54.75%, respectively, while the standard (25 mg/kg) showed writhing and %protection of 7.80 ± 1.32 and 78.21%, respectively (Table 4).

**Table 4:** Effects of *B. pilosa* ethanol extract and controls on acetic acid induced writhing mice

Treatments per oral		Writhing	% protection
NC (10 ml/kg)		35.80 ± 1.07	0
PC (25 mg/kg)		7.80 ± 1.32*	78.21
Extract (mg/kg)	250	24.60 ± 1.21*	31.28
	500	16.20 ± 1.16*	54.75

Values are mean ± SEM (*n* = 5) and percentage; *t*-student’s test at 95% confidence intervals

*NC* negative control (Vehicle), *PC* positive control (Diclofenac sodium)

^*^p < 0.05 when compared to the NC (negative control) group

## Discussion

Solvent extraction is one of the safest methods for the extraction of a wide variety of medicinal plants. High proof alcohols (190 and 210) are used for extraction applications. Ethanol is the emerging and more popular solvent, as it is safe for infusing edibles. It is compatible with any type of container-easily recoverable and it provides consistent results during the evaluation of the crude extract. In this study, we also prepared an extraction with this popularly used solvent.

Inflammation is a major biological process that regulates the interaction between organisms and the environment [8]. Chronic and low-grade inflammation may cause cellular aging and many diseases, including arthritis, allergies, atherosclerosis, and cancer [16]. Tissue protein denaturation may produce auto-antigens in certain arthritic diseases. Therefore, it is a marker for inflammation and arthritic diseases [1]. Protein denaturation results in inflammation through loss of tertiary and secondary structures of proteins from the effects of heat, strong acid/base, inorganic salt or organic solvents [26]. Anti-inflammatory drugs can be used to inhibit protein denaturation [43]. In this study, *B. pilosa* extract showed significant anti-inflammatory activity by inhibiting the heat-induced lysis of egg albumin. Phytochemicals such as alkaloids and flavonoids present in the plant are responsible for giving anti-inflammatory effects [3]. Moreover, terpenoids [18], glycosides and other plant-derived secondary metabolites are the potential sources of anti-inflammatory agents [40]. In the present study, we also found diverse secondary metabolites in *B. pilosa* extract, suggesting a strong foundation for further studies aiming to isolate anti-inflammatory lead compounds from this medicinal herb. In a recent study, the methanol extract has been reported to have a concentration-dependent (62.5 to 500 μg/mL) membrane stabilizing activity [22]. The highest percentage inhibitory observed in the case of ethanol extract in this study is comparable to the highest concentration (320 μg/mL) of methanol extract.

According to the World Health Organization (WHO), approximately 65% of the world’s population uses traditional medicine for medical care [40]. Plants are the reservoirs of potential secondary metabolites (major sources of drugs), that can be used to treat diverse pathological states in humans [53]. Several anti-inflammatory medicinal plant species, such as *Myracroduo nurundeuva* Allemão, *Schinus terebinthifolius* Raddi, *Spondias mombin* L., *Spondias purpurea* L., *Spondias tuberosa* Arruda, *Euphorbiaceae acalypha* hispida Burm. f., *Acalypha indica* L. and *Phyllanthus niruri* L. [4, 9, 38, 47, 54, 61]. The authors reported that it may be due to the presence of phenols, triterpenes, flavonoids, saponins, and tannins in their extracts. The current study also reports that *B.* pilosa ethanol extract also posseses terpenoids, saponins, and tannins. Thus, the anti-inflammatory effects of *B. pilosa* might be due to its above-mentioned secondary metabolites.

Atherothrombosis, characterized by the formation of one or more atheromatous plaques inside the blood vessels, is one of the major consequences of morbidity and mortality worldwide [33, 42]. The fibrinolytic agents are used to lysis the clotted blood inside the vessels [2]. Alteplase, anistreplase, streptokinase, urokinase, and tissue plasminogen (TPA) are also used to treat thrombosis [25]. Among them, streptokinase and urokinase are widely used due to their low cost [10, 39]. Cumulative reports suggest that herbal products have promising anti-thrombotic effects [21, 30]. Plant-derived secondary metabolites such as polyphenols [7], alkaloids [41], and glycosides [63] are evident to possess anti-atherothrombosis activity. Streptokinase is an extracellular fibrinolytic enzyme secreted by the beta-hemolytic *Streptococci* bacteria [31]. It works through binding to both free and fibrin bound plasminogen to form a complex, and subsequently converts the other free plasminogen into an activated protease plasmin, thus dissolving the clotted blood in the blood vessels [35]. The most frequent use of this drug is in the treatment of acute myocardial infarction, deep vein thrombosis, and pulmonary embolism [14]. Our study demonstrates that *B. pilosa* extract has significant concentration-dependent clot lysis capacity. It is to be mentioned that obesity-driven chronic inflammation along with an impaired fibrinolysis event appear to be one of the major effector mechanisms of thrombosis in obesity [6]. One study reports that the methanol extract of *B. pilosa* is evident to exert an anti-obesity effect on *Swiss* albino mice [62]. Therefore, anti-obesity and clot lysis capacity might be related to each other. Sedighi et al. [52] report that medicinal plants are a hope for treating atherosclerosis through reducing cholesterolemia, free radicals, inflammation, vascular resistance, and important enzymes. Thus, the medicinal plant alone or in combination with hypocholesterolemic drugs, can be a useful tool for the treatment of patients with hyperlipidemia and its complications. In a study, zinc chelation was found to promote streptokinase-induced thrombolysis in vitro [58].

Acetic acid causes the release of several endogenous substances (e.g., serotonin, bradykinins, histamine, prostaglandins (PGs), substance P) that are liable to cause pain by accelerating nerve endings. Locally sensitized peritoneal receptors are responsible for the activation of abdominal constrictions response [48]. This model has also been connected with prostanoids that increase the levels of PG-E_2_ and -E_2α_ in peritoneal fluids, as well as lipoxygenase products [60]. In this study, *B. pilosa* extract was found to exhibit a significant anti-nociceptive effect on test animals. The inhibition of synthesis and release of PGs and other endogenous substances may be the most probable pathway for peripherally acting analgesics. Jyoti et al. [22] have reported a dose-dependent analgesic effect in the formalin-induced paw licking mouse model. Our study also demonstrates a dose-dependent anti-nociceptive effect in an acetic acid induced writhing mouse model. In both cases, the activity observed is comparable. It seems our reports agree with the findings of Jyoti et al. [22].

The analgesic profiles of many plant species are widely used in folk medicine, for example, *Hyptis pectinata* Poit. (Lamiaceae) [5], *Erythrina velutina* Willd. (Fabaceae) [11], and *Hyptis fruticosa* Salzm. ex Benth. (Lamiaceae) [37]. These plants are evident to contain terpenoids. Thus, terpenoids may be important tools for pain management in animals. In general, pain is an unpleasant sensation that is associated with real or potential tissue damage. The experience of pain is generated in the early stages by direct activation of the sensory nerve fibers. The discomfort in the late phase, on the other hand, is related to inflammatory mediators. Current anti-inflammatory and pain medications are helpful, but they have serious side effects such as ulcers, anemia, osteoporosis, and endocrine disruption. Consequently, there has been a greater emphasis on the investigation of the analgesic potential of phytoconstituents, or traditional medicinal plants, because they frequently exhibit adequate biological activity with fewer side effects when compared to manufactured medicines [24]. The current study demonstrates that 500 mg/kg (p.o.) of *B.* pilosa ethanol extract showed a significant analgesic effect on the experimental animal. Thus, the potent anti-inflammatory and analgesic effects of its highest concentration/dose may co-relate with each other.

Our study reports that *B. pilosa* contains many important secondary metabolites. The practice of treating diseases by using medicinal plants and their derivatives dates back to ancient civilizations. Plant secondary metabolites are good sources for treating diverse ailments (Hu et al., 20,020). Among the secondary metabolites, terpenoids, phenolics, flavonoids, alkaloids and glycosides are known as important ingredients in nutraceuticals as well as modern medicines [49, 59]. Generally, most secondary metabolites have potent antioxidant properties. Therefore, they have protective capacity [44]. These metabolites are evident to impart a wide range of therapeutic activities; they can directly interact with receptors, cell membranes, and genetic materials (e.g., DNA, RNA) [57].

Risk/benefit evalation of a herbal product is crucial. For this, pre-clinical and non-clinical studies play important roles in the study of the safety potential of a wide variety of crude drugs of natural origins. Experimental animals such as mice and rats are frequently used for this purpose [36]. According to Jyoti et al. [22], the methanol extract up to 3000 mg/kg (p.o.) of this herb did not show acute toxicity in *Swiss* mice. It seems the extract is well-tolerated in *Swiss* mice.

## Conclusion

Our study suggests that *B. pilosa* possesses many important secondary metabolites such as alkaloids, flavonoids, glycosides, terpenoids, tannins, saponins, and reducing sugars. *B. pilosa* has shown significant thrombolytic, anti-inflammatory, and anti-nociceptive activities on the test systems applied in our study. *B. pilosa* might be a hopeful alternative medicinal plant to fight against various diseases, including atherothrombosis, inflammation and inflammatory diseases. However, the molecular interactions between the phytochemicals of this herb and the observed biological activities in this study are yet to be determined. Therefore, further studies are necessary to isolate the lead compounds from the hopeful medicinal herb and evaluate the possible mechanism (s) for each pharmacological effect by adopting suitable test models.

## Data Availability

Not applicable.

## References

[1] Al Nayeem A, Khatun A, Rahman MS, Rahman M (2011). Evaluation of phytochemical and pharmacological properties of *Mikania cordata (Asteraceae)* leaves. J Pharmacogn Phytother.

[2] Banerjee A, Chisti Y, Banerjee UC (2004). Streptokinase-a clinically useful thrombolytic agent research review paper. Biotechnol Adv.

[3] Banerjee S, Chanda A, Adhikari A, Das AK, Biswas S (2014). Evaluation of phytochemical screening and anti inflammatory activity of leaves and stem of *Mikania scandens* (L.) wild. Ann Med Health Sci Res.

[4] Barbosa HM, Nascimento JN, Araújo TA, Duarte FS, Albuquerque UP, Vieira JR, Santana ER, Yara R, Lima CS, Gomes DA, Lira EC (2016). Acute toxicity and cytotoxicity effect of ethanolic extract of *Spondias tuberosa* arruda bark: hematological, biochemical and histopathological evaluation. An Acad Bras Cienc.

[5] Bispo MD, Mourão RH, Franzotti EM, Bomfim KB, Arrigoni-Blank MF, Moreno MP, Marchioro M, Antoniolli AR (2001). Antinociceptive and antiedematogenic effects of the aqueous extract of *Hyptis pectinata* leaves in experimental animals. J Ethnopharmacol.

[6] Blokhin IO, Lentz SR (2013). Mechanisms of thrombosis in obesity. Curr Opin Hematol.

[7] Booyse FM, Pan W, Grenett HE, Parks DA, Darley-Usmar VM, Bradley KM, Tabengwa EM (2007). Mechanism by which alcohol and wine polyphenols affect coronary heart disease risk. Ann Epidemiol.

[8] Bordoni A, Danesi F, Dardevet D, Dupont D, Fernandez AS, Gille D, Nunes Dos Santos C, Pinto P, Re R, Rémond D, Shahar DR, Vergères G (2017). Dairy products and inflammation: a review of the clinical evidence. Crit Rev Food Sci Nutr.

[9] Cabral B, Siqueira EMS, Bitencourt MAO, Lima MCJS, Lima AK, Ortmannd CF, Chaves VC, Fernandes-Pedrosa MF, Rochac HAO, Scortecci KC (2016). Phytochemical study and anti-inflammatory and antioxidant potential of *Spondias mombin* leaves. Rev Bras.

[10] Collen D (1990). Coronary thrombolysis: streptokinase or recombinant tissue-type plasminogen activator?. Ann Intern Med.

[11] Dantas MC, De Oliveira FS, Bandeira SM, Batista JS, Silva CD, Alves PB, Antoniolli AR, Marchioro M (2004). Central nervous system effects of the crude extract of *Erythrina velutina* on rodents. J Ethnopharmacol.

[12] Decker EL, Reski R (2012). Glycoprotein production in moss bioreactors. Plant Cell Rep.

[13] Di Lorenzo C, Ceschi A, Kupferschmidt H, Lüde S, De Souza NE, Dos Santos A, Colombo F, Frigerio G, Nørby K, Plumb J, Finglas P (2015). Adverse effects of plant food supplements and botanical preparations: a systematic review with critical evaluation of causality. Br J Clin Pharmacol.

[14] Dundar Y, Hill R, Dickson R, Walley T (2003). Comparative efficacy of thrombolytics in acute myocardial infarction: a systematic review. QJM..

[15] Ezeja M, Omeh Y, Ezeigbo I, Ekechukwu A (2011). Evaluation of the analgesic activity of the methanolic stem bark extract of *Dialium guineense* (wild). Ann Med Health Sci Res.

[16] Franceschi C, Campisi J (2014). Chronic inflammation (inflammaging) and its potential contribution to age-associated diseases. J Gerontol A Biol Sci Med Sci.

[17] Ghani A (2003). Medicinal plants of Bangladesh.

[18] González Y, Torres-Mendoza D, Jones GE, Fernandez PL (2015). Marine Diterpenoids as potential anti-inflammatory agents. Mediat Inflamm.

[19] Harborne JB (1984). Phytochemical methods - a guide to modern techniques of plant analysis.

[20] Hong L, Guo Z, Huang K, Wei S, Liu B, Meng S, Long C (2015). Ethnobotanical study on medicinal plants used by Maonan people in China. J Ethnobiol Ethnomed.

[21] Huang Y, Tsang SY, Yao X, Chen ZY (2005). Biological properties of baicalein in cardiovascular system. Curr Drug Targets Cardiovasc Haematol Disord.

[22] Jyoti MA, Barua N, Hossain MS, Hoque M, Bristy TA, Mahmud S, Kamruzzaman AM, Chy MNU, Paul A, Hossain ME, Emran TB, Simal-Gandara J (2020). Unravelling the biological activities of the *Byttneria pilosa* leaves using experimental and computational approaches. Molecules.

[23] Khadka D, Dhamala MK, Li F, Aryal PC, Magar PR, Bhatta S, Thakur MS, Basnet A, Cui D, Shi S (2021). The use of medicinal plants to prevent COVID-19 in Nepal. J Ethnobiol Ethnomed.

[24] Khan H, Pervaiz A, Intagliata S, Das N, Venkata KCN, Atanasov AG, Najda A, Nabavi SM, Wang D, Pittalà V, Bishayee A (2020). The analgesic potential of glycosides derived from medicinal plants. Daru.

[25] Khan IN, Habib MR, Rahman MM, Mannan A, Sarker MMI, Hawlader S (2010). Thrombolytic potential of *Ocimum sanctum* L.*, Curcuma longa* L.*, Azadirachta indica* L. and *Anacardium occidentale* L. J Basic Clin Pharm.

[26] Khandia R, Munjal AK, Iqbal HMN, Dhama K (2017). Heat shock proteins: therapeutic perspectives in inflammatory disorders. Recent Patents Inflamm Allergy Drug Discov.

[27] Koster R, Anderson M, De Beer EJ (1959). Acetic acid for analgesic screening. Fed Proc.

[28] Kubinyi H (1999). Chance favors the prepared mind-from serendipity to rational drug design. J Recept Sig Transd.

[29] Lautié E, Russo O, Ducrot P, Boutin JA. Unraveling plant natural chemical diversity for drug discovery purposes. Front Pharmacol. 2020. 10.3389/fphar.2020.00397. 10.3389/fphar.2020.00397 PMC7154113 32317969

[30] Majewski M (2014). *Allium sativum*: facts and myths regarding human health. Rocz Panstw Zakl Hig.

[31] Malke H, Ferretti JJ (1984). Streptokinase: cloning, expression, and excretion by Escherichia coli. Proc Natl Acad Sci U S A.

[32] Margolin E, Chapman R, Williamson A, Rybicki EP, Meyers AE (2018). Production of complex viral glycoproteins in plants as vaccine immunogens. Plant Biotechnol J.

[33] Martin-Ventura JL, Rodrigues-Diez R, Martinez-Lopez D, Salaices M, Blanco-Colio LM, Briones AM. Oxidative stress in human atherothrombosis: sources, markers and therapeutic targets. Int J Mol Sci. 2017;18(11). 10.3390/ijms18112315. 10.3390/ijms18112315 PMC5713284 29099757

[34] McAlpine JB, Bachmann BO, Piraee M, Tremblay S, Alarco AM, Zazopoulos E, Farnet CM (2005). Microbial genomics as a guide to drug discovery and structural elucidation: ECO-02301, a novel antifungal agent, as an example. J Nat Prod.

[35] McClintock DK, Bell PH (1971). The mechanism of activation of human plasminogen by streptokinase. Biochem Biophys Res Commun.

[36] Mei N, Guo X, Ren Z, Kobayashi D, Wada K, Guo L (2017). Review of *Ginkgo biloba*-induced toxicity, from experimental studies to human case reports. J Environ Sci Health C Environ Carcinog Ecotoxicol Rev.

[37] Menezes IA, Marques MS, Santos TC, Dias KS, Silva AB, Mello IC, Lisboa AC, Alves PB, Cavalcanti SC, Marçal RM, Antoniolli AR (2007). Antinociceptive effect and acute toxicity of the essential oil of *Hyptis fruticosa* in mice. Fitoterapia..

[38] Mostofa R, Ahmed S, Begum MM, Sohanur Rahman M, Begum T, Ahmed SU, Tuhin RH, Das M, Hossain A, Sharma M, Begum R (2017). Evaluation of anti-inflammatory and gastric anti-ulcer activity of *Phyllanthus niruri* L. (Euphorbiaceae) leaves in experimental rats. BMC Complement Altern Med.

[39] Mucklow JC (1995). Thrombolytic treatment – streptokinase is more economical than Alteplase. BMJ..

[40] Nunes CR, Arantes MB, Pereira SMF, Cruz LL, Passos MS, Moraes LP, Vieira IJC, Oliveira DB (2020). Plants as sources of anti-inflammatory agents. Molecules.

[41] Olatunji LA, Michael OS, Adeyanju OA, Areola ED, SoladoyeAO. (2017). Anti-inflammatory and antithrombotic effects of nicotine exposure in oral contraceptive-induced insulin resistance are glucocorticoid-independent. Pharmacol Rep.

[42] Pant S, Deshmukh A, Gurumurthy GS, Pothineni NV, Watts TE, Romeo F, Mehta JL (2014). Inflammation and atherosclerosis--revisited. J Cardiovasc Pharmacol Ther.

[43] Patel D, Desai S. Phytochemical screening*, in vitro* anti-microbial and anti-inflammatory activity of methanolic extract of *aster lanceolatus willd* leaves. Int J Med Res. 2016;1(1):26–30.

[44] Pisoschi AM, Pop A, Cimpeanu C, Predoi G (2016). Antioxidant capacity determination in plants and plant-derived products: a review. Oxidative Med Cell Longev.

[45] Prasad S, Kashyap RS, Deopujari JY, Purohit HJ, Daginawala TGM, HF. (2006). Development of an in vitro model to study clot lysis activity of thrombolytic drugs. Thromb J.

[46] Reski R, Parsons J, Decker EL (2015). Moss-made pharmaceuticals: from bench to bedside. Plant Biotechnol J.

[47] Rosas EC, Correa LB, Pádua Tde A, Costa TE, Mazzei JL, Heringer AP, Bizarro CA, Kaplan MA, Figueiredo MR, Henriques MG (2015). Anti-inflammatory effect of *Schinus terebinthifolius* Raddi hydroalcoholic extract on neutrophil migration in zymosan-induced arthritis. J Ethnopharmacol.

[48] Sadhu SK, Okuyama E, Fujimoto H, Ishibashi M (2003). Seperartion of *Leucas aspara*, a medicinal plant of Bangladesh, guided by prostaglandin inhibitory and anti-oxidant activities. Chem Pharm Bull (Tokyo).

[49] Salem MA, de Souza LP, Serag A, Fernie AR, Farag MA, Ezzat SM, Alseekh S (2020). Metabolomics in the context of plant natural products research: from sample preparation to metabolite analysis. Metabolites.

[50] Sánchez-Medina A, García-Sosa K, May-Pat F, Peña-Rodríguez LM (2001). Evaluation of biological activity of crude extracts from plants used in Yucatecan traditional medicine part I. antioxidant, antimicrobial and beta-glucosidase inhibition activities. Phytomedicine.

[51] Sasidharan S, Chen Y, Saravanan D, Sundram KM, Latha LY (2011). Extraction, isolation and characterization of bioactive compounds from plants’ extracts. Afric J Trad Complement Altern Med.

[52] Sedighi M, Bahmani M, Asgary S, Beyranvand F, Rafieian-Kopaei M (2017). A review of plant-based compounds and medicinal plants effective on atherosclerosis. J Res Med Sci.

[53] Shah B, Seth A, Maheshwari K (2011). A review on medicinal plants as a source of anti-inflammatory agents. Res J Med Plant.

[54] Siraj MA, Shilpi JA, Hossain MG, Uddin SJ, Islam MK, Jahan IA, Hossain H (2016). Anti-inflammatory and antioxidant activity of acalypha hispida leaf and analysis of its major bioactive polyphenols by HPLC. Adv Pharm Bull.

[55] Trease G, Evans SM (2002). Pharmacognosy.

[56] Ullah HMA, Zaman S, Juhara F, Akter L, Tareq SM, EH, Bhattacharjee R. (2014). Evaluation of antinociceptive, in-vivo & in-vitro anti-inflammatory activity of ethanolic extract of *Curcuma zedoaria* rhizome. BMC Complement Altern Med.

[57] Velu G, Palanichamy V, Rajan AP, Roopan S, Madhumitha G (2018). Phytochemical and pharmacological importance of plant secondary metabolites in modern medicine. Bioorganic phase in natural food: an overview.

[58] Wang Z, Yu X, Li YV (2017). Zinc chelation promotes streptokinase-induced thrombolysis *in vitro*. Int J Physiol Pathophysiol Pharmacol.

[59] Wink M (2015). Modes of action of herbal medicines and plant secondary metabolites. Medicines (Basel).

[60] Yu Y, Jia TZ, Cai Q, Jiang N, Ma MY, Min DY, Yuan Y (2015). Comparison of the anti-ulcer activity between the crude and bran-processed Atractylodeslancea in the rat model of gastric ulcer induced by acetic acid. J Ethnopharmacol.

[61] Zahidin NS, Saidin S, Zulkifli RM, Muhamad II, Ya'akob H, Nur H (2017). A review of *Acalypha indica* L. (Euphorbiaceae) as traditional medicinal plant and its therapeutic potential. J Ethnopharmacol.

[62] Zaman R, Parvez M, Ali MS, Islam M (2015). Possible anti-obesity activity of methanol extract of *Byttneria pilosa* Roxb. leaves. Middle-East J Sci Res.

[63] Zhu X, Fang ZH (2014). Newmonoterpene glycosides from the root cortex of *Paeonia suffruticosa* and their potential anti-inflammatory activity. Nat Prod Res.

